# Metastatic Klebsiella Pneumoniae in an Immunocompetent Patient: A Rare, Atypical Presentation of Klebsiella Syndrome

**DOI:** 10.7759/cureus.26156

**Published:** 2022-06-21

**Authors:** Eric Y Choi, Abdul Waheed, Siamak M Seraj

**Affiliations:** 1 Family Medicine, Campbell University, Conway Medical Center, Conway, USA; 2 Surgery, San Joaquin General Hospital, San Joaquin, USA; 3 Internal Medicine, San Joaquin General Hospital, San Joaquin, USA

**Keywords:** bacterial liver abscess, endophthalmitis, immunocompetent patients, pneumoniae, klebsiella

## Abstract

Metastatic *Klebsiella pneumoniae* (MKP) is a rare, atypical presentation of Klebsiella syndrome. The disease primarily affects patients with underlying immunocompromised status, but its prevalence in immunocompetent patients without any underlying illness is rare. We present a rare case of MKP in a 41-year-old Caucasian male without prior comorbidities who presented with blurry vision and was found to have MKP. The current case report also discusses the diagnostic modalities, complications, and treatment options of MKP.

## Introduction

*Klebsiella pneumoniae* is an anaerobic, Gram-negative bacterium that can cause pneumonia, meningitis, and liver abscess, among other life-threatening illnesses [1]. Both in the community and the hospital, these infections are common [2]. Like most infections, the underlying immunocompromised status is vital in disseminating the disease, mainly metastatic *Klebsiella pneumoniae* (MKP). The prevalence of the MKP in immunocompromised patients has been reported, but the prevalence in immunocompetent individuals is a rare phenomenon [1,2].

Additionally, the MKP has gotten much attention in eastern countries over the previous decade. Even with broad-spectrum medicines, the MKP fatality rate has risen to 60% because of antibiotic-resistant bacteria [3,4]. In addition, Klebsiella develops a metastatic disease in typical community-acquired situations and presents itself predominantly as a monomicrobial disease [1,5]. The current case report described a rare case of MKP and aimed to elaborate on the presentations, diagnostic modalities, complications, and current management options.

## Case presentation

A 41-year-old Caucasian male with no significant medical history presented with abdominal pain to the emergency room for one week. He described having severe pain in the right upper quadrant radiating to the left flank. He also reported subjective fevers, chills, nausea, and decreased appetite. Two days after the initial symptoms, the patient noticed the blurry vision in his left eye, prompting him to seek medical attention. On admission to the emergency room, his vital signs were unremarkable. A physical examination revealed left conjunctival hyperemia, absent red reflex, and painful ocular movements, which limited further eye examination. Diffuse tenderness to palpation in the abdomen, especially in the right upper quadrant, was elicited along with left costovertebral angle tenderness.

**Figure 1:**
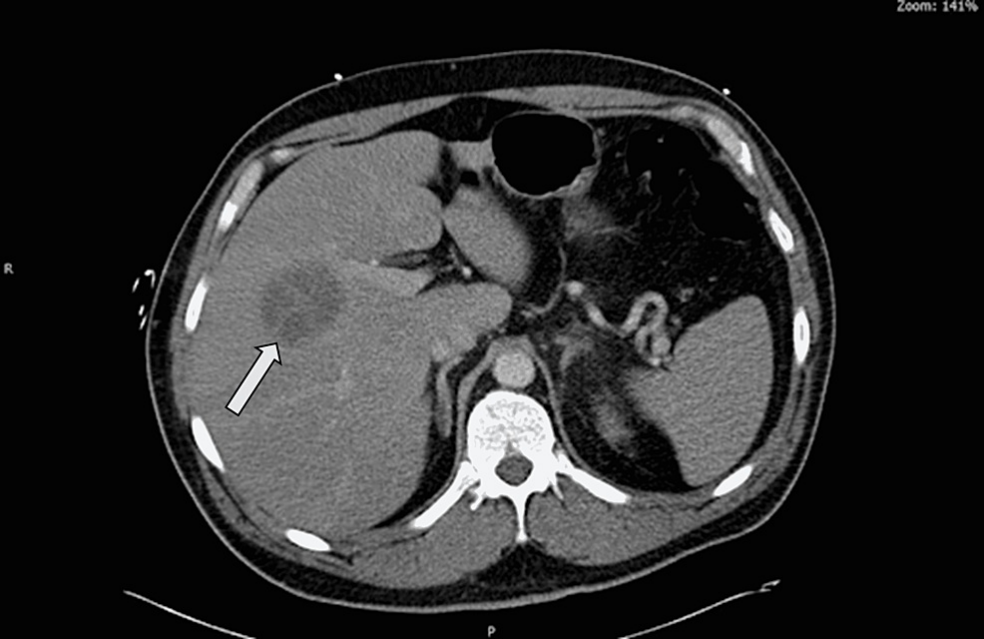
Computed tomography (CT) scan of abdomen and pelvis showing a septated hypodense lesion of 4.8 x 4.5 cm in central liver (arrow).

Lab findings were significant for white blood cell (WBC) count of 18.4 µL, serum creatinine 1.08 mg/dL (0.5-1.4 mg/dL), total bilirubin of 5.5 mg/dL (0.2-1.2 mg/dL), aspartate transaminase (AST) 76 unit/L (5-34 unit/L), alanine transaminase (ALT) 87 unit/L (0-55 unit/L), and alkaline phosphatase (ALP) 368 unit/L (40-150 unit/L). Urinalysis was positive for leukocytes, nitrites, urobilinogen, bilirubin, WBCs, and bacteria. The computed tomography (CT) scan of the abdomen and pelvis obtained in the emergency department showed decreased enhancement and surrounding inflammatory changes in the upper pole of the left kidney, likely representing pyelonephritis and a low-density mass likely secondary to an abscess. A septate hypodense lesion in the central liver was also seen, measuring approximately 4.8 x 4.5 cm transversely and 6.0 cm craniocaudal in diameter, possibly raising concern for a liver abscess (Figure 1).

**Figure 2:**
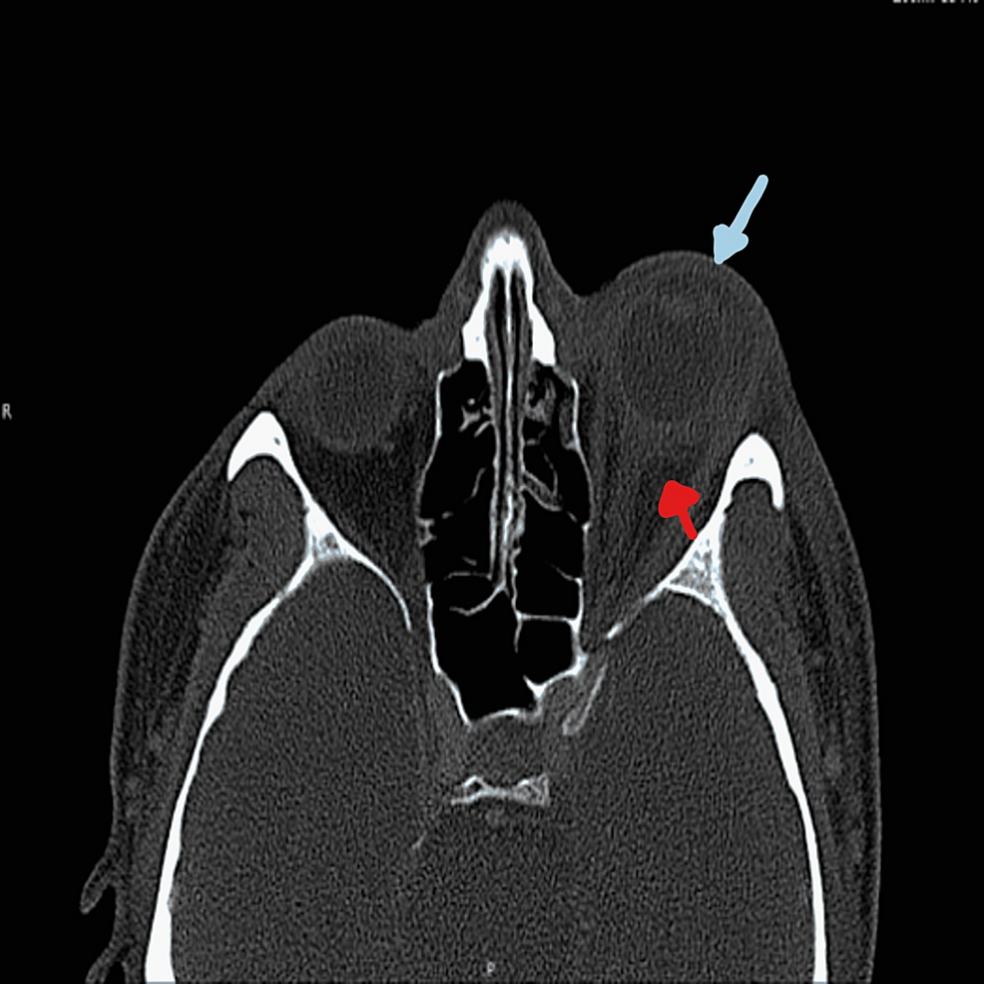
CT scan (skull) of facial bones and periorbital sinuses (blue and red arrows).

**Figure 3:**
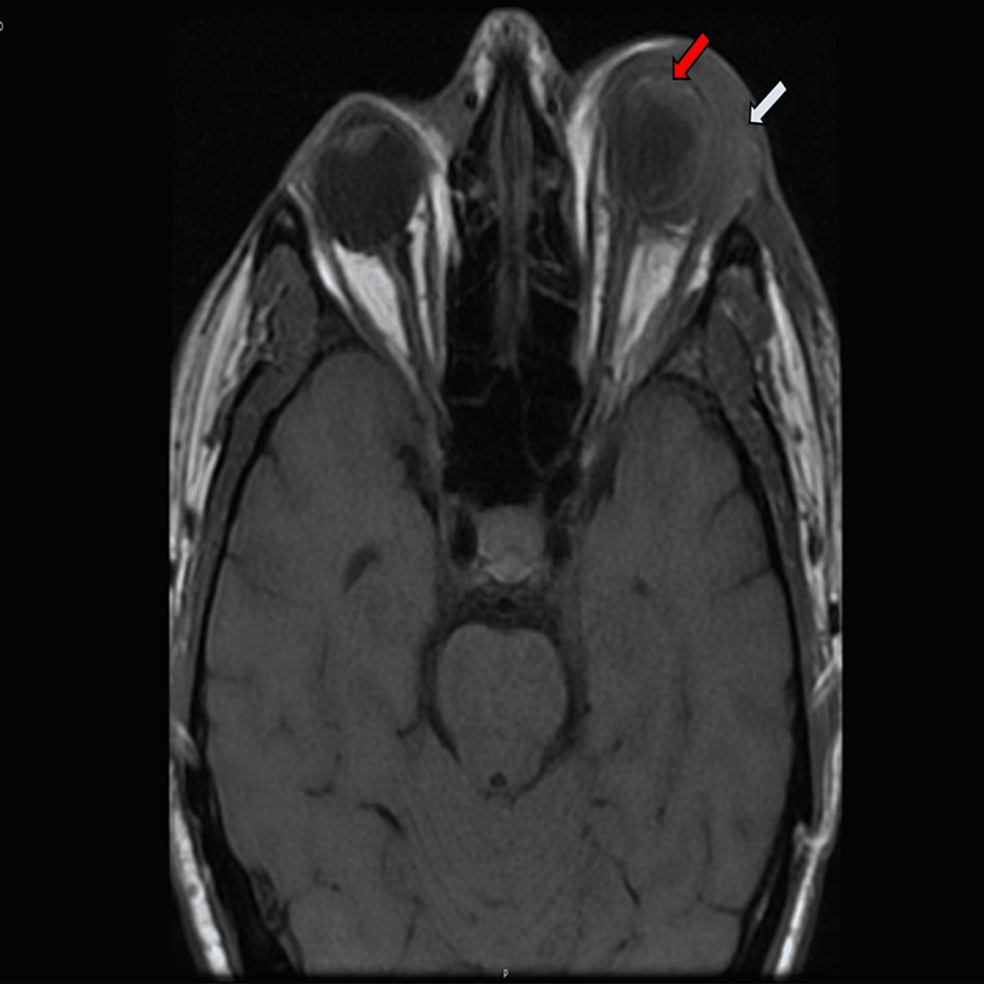
MRI of head showing (a) preseptal edema; white arrow and (b) periorbital edema; red arrow.

The patient was admitted for the inpatient care, and on hospital day two, the patient was found to have a blood culture and urine culture positive for *Klebsiella pneumoniae*. The patient continued to have persistent eye pain. Upon further investigation, the magnetic resonance imaging (MRI) of the head and CT scan of facial bones revealed circumferential preseptal and postseptal edema compatible with endophthalmitis (Figures 2, 3). A non-drainable fluid collection was also noted to be suggestive of a phlegmon.

Otolaryngology and ophthalmology services were consulted. The ophthalmologist conducted an eye examination showing minimal light perception in the left eye without a discernible view of the retina. The slit-lamp test showed visible purulent matter in the inferior and anterior chamber, most consistent with a hypopyon. The right eye examination was unremarkable. Due to his active infection and already lost vision, the patient was not considered a favorable candidate for surgical vitrectomy or vitreous antibiotic injections. Klebsiella was fully sensitive to all routine antibiotics. The patient was placed on IV ceftriaxone 2 g daily for six weeks. Following IV antibiotic therapy our patient required extra four months of oral antibiotics with ciprofloxacin 500 mg BID as there was sign of residual abscess in the liver in follow-up CT scans.

Four weeks after discharge, the patient followed up with the infectious disease team. Blood cultures were negative, and the liver abscess responded to continued antibiotic therapy. Repeat imaging showed a decrease in size from 4.1 cm to 2.5 cm, and the patient was started on oral ciprofloxacin 500 mg twice a day and intravenous ceftriaxone was given as the patient already devolved dissemination and endophthalmitis. Studies have shown that minimal inhibitory concentration in aqueous humor can be adequately achieved if antibiotic combination is used. He was also seen in the oculoplastic surgery clinic regarding the intervention of his now resolved endophthalmitis with the recommendation of enucleation at a future date. At his eight-week follow-up, repeat imaging showed a persistent liver mass with mild improvement in size from 2.5 cm to 1.9 cm. To the best of our knowledge, no authentic data exists supporting the use of ciprofloxacin 750 mg BD. Patient responded to long duration of ciprofloxacin 500 mg BID. In this unfortunate case, vision loss had already occurred and organ-saving measures were not focus of treatment anymore so ciprofloxacin 500 mg orally twice a day was recommended for two weeks with repeat imaging to evaluate abscess resolution.

## Discussion

*Klebsiella pneumoniae* is one of the many clinically virulent species of the Klebsiella genus [5]. It is an important pathogen causing hospital-acquired septicemia and is associated with the emergence of community-acquired pyogenic liver abscess (PLA) [6]. In a case-control study of 117 patients done by Hansen et al., *Klebsiella pneumoniae* bacteremia was more often found in males, associated with recent hospital contact within the previous month, polymicrobial infection, recent surgery, received steroid therapy, invasive plastic devices, and diabetes mellitus. The most common source of Klebsiella bacteremia was urinary tract infection, followed by biliary tract infection. However, 27% of cases in this study had no source of disease [5].

In a case series by Wang et al. reviewing 182 reports, the most common clinical features of systemic *K. pneumoniae* infection presentation included fever, leukocytosis, elevated serum alanine and aspartate aminotransferases, elevated alkaline phosphatase, and right upper quadrant tenderness [6]. This array of symptoms is often non-specific and seen in other polymicrobial etiologies of hepatic abscesses. Abdominal ultrasound and computer tomography scans are non-specific and show either unilobular or multilobular masses; however, *K. pneumoniae* liver abscesses often appear more solid with less purulent aspirate than other etiologies [7-12]. As patients with liver abscesses have concurrent bacteremia, the definitive diagnosis can be made with positive blood or aspirate culture [1,13-15]. As reported by Ko et al. review of 455 patients with bacteremia, only four community-acquired bacteremia *K. pneumoniae *pneumonia cases were seen in a two two-year study period. Twenty-five cases of a distinctive syndrome include *K. pneumoniae* bacteremia associated with liver abscess, meningitis, or endophthalmitis [2].

Moreover, in a retrospective analysis of 18 cases of Klebsiella liver abscess (KLA) done by Lederman et al., 83% had concurrent bacteremia, and 28% had accompanying metastatic complications. In recent years, *K. pneumoniae *has been the most common pathogen causing community-acquired pyogenic hepatic abscesses [1, 3,13]. These intra-abdominal infections have the highest incidence in East Asia, particularly in Taiwan, but reports of *K. pneumoniae* liver abscesses have increased worldwide. Therefore, the diagnosis of *K. pneumoniae* should be considered in all cases of PLA [3]. Although rare, systemic bacteremia caused by PLA can lead to metastatic infection. In a review of 23 cases of PLA with septic metastatic lesions and 164 cases of PLA without septic metastatic lesions, KLA, bacteremia, and diabetes mellitus were significantly more common in the study group than in the comparison group. The most common sites of infectious seeding are endogenous endophthalmitis or uveitis (60%), pulmonary abscesses (43.4%), brain abscess (26%), bacteriuria and prostate abscess (21.7%), osteomyelitis (8.6%), and psoas abscess (4.3%) [2,9,12,13].

Additionally, the pathogenies of MKP are poorly understood, yet the precise subtype leading to the disseminated infection has been reported in the previous literature. Pneumoniae K1 genotype was an emerging pathogen capable of causing ocular or central nervous system complications compared to the K2 genotype [1]. In a review of three case series with 524 patients, 12% of patients exhibited concurrent metastatic infection, with 6% having endophthalmitis. Furthermore, similar to the findings in our case report, a study conducted on 61 patients by Ang et al. found that the most common symptom of MLP includes ocular pain, poor vision, hypopyon, and conjunctival erythema The patients who presented with hypopyon and unilateral involvement had a poorer prognosis [4,8]. Additionally, due to the non-specific symptoms, endogenous endophthalmitis is often misdiagnosed or undertreated; *K. pneumoniae* is known to be rapidly progressing and may lead to permanent structural damage and vision loss if not treated urgently [16]. In a retrospective review of 289 patients with endogenous endophthalmitis from metastatic *K. pneumoniae*, a poorer prognosis was seen in patients that were not diagnosed and treated within 24 hours of symptom onset [16].

Also, underlying comorbidities play a significant role in disseminating the disease. Diseases like diabetes mellitus or hepatobiliary involvement increase the risk for rapid infection and less favorable outcomes in patients with endogenous endophthalmitis [17]. Aggressive therapy, including early vitrectomy with antibiotic injection, may improve outcomes. Early detection, aggressive antibiotic treatment, and ophthalmology service consultation are recommended within 24 hours of presentation to prevent misdiagnosis and permanent visual loss [18]. Furthermore, patients with impaired visual acuity on presentation are more likely to have permanent visual deficits even after completing an entire course of antibiotics when followed up at three months postinfection [8]. Similarly, the early detection and treatment of *K. pneumoniae* are essential in preventing the progression of pyogenic liver and metastatic infections. Antibiotic management can be chosen from culture sensitivities.

Ciprofloxacin and moxifloxacin have been used as adjunctive systemic antibiotics for Klebsiella endophthalmitis. In addition, it has been reported that an appropriate minimum inhibitory concentration (MIC) in the aqueous and vitreous humor can be achieved with fluoroquinolones 500 mg when these antibiotics are administered orally or applied topically every two hours. The maximum intraocular concentrations are obtained within 24-48 hours. However, fluoroquinolones can cause harm to the tendons, muscles, joints, nerves, and CNS.

There is no uniform consensus on the maximum dose of the antibiotic for the metastatic *K. pneumoniae* infection and selection is based on a case-by-case basis. A cephalosporin-based antibiotic is the most commonly used antibiotic to treat a *K. pneumoniae* abscess. It is more effective to use third-generation cephalosporins than first-generation cephalosporins in treating infections. Only a small amount of data supports using first-generation cephalosporins to prevent mortality and other adverse effects. Other cephalosporins have been discovered to have a decreased risk of metastatic infection. Hepatic abscesses can be treated with antibiotics such as ampicillin-sulbactam, aztreonam, and quinolones. The use of aminoglycosides, notwithstanding their inability to penetrate abscesses, is prevalent in the medical community. Aminoglycosides have been shown to be toxic, which may negate this advantage. Intravenous antibiotics, as opposed to those taken orally, are only effective for a few weeks. It may be required for some individuals to undergo a longer course of treatment if they have several drainage procedures or radiographic indications of the abscess. Most abscesses are self-healing; however, they may recur even if prescribed medications. Imaging studies and re-aspiration should be performed if a patient has a persistent fever or stomachache. Patients' health improves because of draining abscesses. Removing multiple *K. pneumoniae* abscesses from a single patient may not be possible as soon as they are discovered. To remove an infection, introduce a drain into the infected location and wait for it to mature [15-19]. A carbapenem may be added when faced with extended-spectrum beta-lactamases (ESBL) resistant strains of Klebsiella. According to a prospective study of 455 patients, 85 episodes were due to ESBL *K. pneumoniae, *and carbapenem (imipenem) administration five days after onset of bacteremia was independently associated with lower mortality [19].

For *K. pneumoniae* pyogenic liver abscesses, treatment follows guidelines for standard liver abscesses with percutaneous drainage (percutaneous needle aspiration and continuous percutaneous catheter drainage) when possible and systemic antibiotics until cultures results are obtained [9,19]. Therapy duration may depend on abscess size and metastatic progression. Standard management ranges from four to six weeks with regular follow-up to monitor the complete resolution of abscess on imaging. Metastatic infection, mainly endophthalmitis, requires additional vitreous taps and intravitreal antibiotics to prevent deterioration. More invasive options such as enucleation and evisceration may be considered if conventional medical treatment fails. Endophthalmitis caused by Klebsiella species is associated with poor visual outcomes and has high rates of enucleation or evisceration [4,19].

In this case, the patient did not have any identifiable risk factors which could exacerbate the pathogenies of such an aggressive infection.​​​​ He was a previously healthy Caucasian male without recent travel domestically or internationally. His past medical history was unremarkable and without any comorbid conditions, including diabetes, cirrhosis, hepatobiliary disease, malignancy, or any chronic, immunocompromised diseases. In addition, he had no recent hospitalizations or antibiotic use to lead him to have a higher susceptibility to *K. pneumoniae *septicemia or its severe progression. Our case exhibits rare, atypical epidemiology of a classic *K. pneumoniae* infection and the rapidly progressing complications in a previously healthy Caucasian male.

## Conclusions

MKP in immunocompetent patients is an infrequent phenomenon. Early treatment with mono or dual antibiotics may reduce infection duration, hepatic abscess size, and metastatic rate. The patient reported vision deterioration and non-specific septicemia symptoms in the current case report. *K. pneumoniae* liver abscess syndrome is widespread in Asian patients with diabetes or hepatobiliary illness. As our case shows,* K. pneumoniae* infection and liver abscess syndrome should be evaluated in the differential diagnosis of any patient with this constellation of unspecific clinical symptoms. In contrast to candidemia, there are no proven guidelines that are advised for the screening of patients who are at high risk for metastatic infection. Patients diagnosed with Klebsiella septicemia and liver abscess should strongly consider having an eye examination performed by an ophthalmologist, based on our experiences with this case. Vision and meningeal sign examinations should be advised in all Klebsiella septicemias irrespective of their sensitivity to antibiotic analysis regimen.
